# Combined microscope–endoscopy resection of petrous bone cholesteatoma with temporary facial nerve transposition versus nontransposition

**DOI:** 10.1007/s00405-023-08397-9

**Published:** 2024-01-16

**Authors:** Honglin Mei, Xiaoling Lu, Chunguang Dong, Hailiang Lin, Bing Chen, Huawei Li, Yusu Ni

**Affiliations:** 1 Department of Otorhinolaryngology, Eye and ENT Hospital, ENT Institute, Fudan University, Shanghai, 200031 China; 2 NHC Key Laboratory of Hearing Medicine, Fudan University, Shanghai, 200031 China; 3 Department of Otorhinolaryngology, First People’s Hospital of Lianyungang, Jiangsu, 222002 China; 4 Institutes of Biomedical Sciences, Fudan University, Shanghai, 200032 China; 5 The Institutes of Brain Science and the Collaborative Innovation Center for Brain Science, Fudan University, Shanghai, 200032 China; 6 Otology and Skull Base Surgery, Eye and ENT Hospital, Fudan University, Shanghai, 200031 China

**Keywords:** Cholesteatoma, Petrous bone, Facial nerve transposition, Microscopic surgery, Endoscopic surgery

## Abstract

**Purpose:**

The narrow supralabyrinthine space affects surgical procedures. To study the effect of temporary transposition of geniculate ganglion of facial nerve versus nontransposition on lesion recurrence and facial nerve function in patients with petrous bone cholesteatoma.

**Methods:**

A total of 18 patients with petrous bone cholesteatoma involving the facial nerve were treated in our hospital from November 2016 to March 2023. The main surgical method is the extended supralabyrinthine approach assisted by a microscope and an endoscope. We collected and retrospectively analyzed their medical records.

**Results:**

Temporary facial nerve transposition was performed in five patients, and nontransposition was performed in 13 patients. Cholesteatoma recurred in three patients with facial nerve nontransposition, whereas none in patients with facial nerve transposition. In this study, except for one case with a second operation, postoperative facial paralysis in other cases was improved to varying degrees, and there was no significant difference between the two groups.

**Conclusion:**

Temporary transposition of geniculate ganglion of facial nerve will not affect the postoperative nerve function of patients and can reduce the possibility of cholesteatoma recurrence of the petrous bone.

## Introduction

Petrous bone cholesteatoma refers to cholesteatoma medial to the otic capsule. It has slow growth, a complex location in the skull base, and proximity to important neurovascular structures, which make its diagnosis and treatment very challenging [1].

Once petrous bone cholesteatoma is found, it should be treated immediately. The selection of surgical approaches needs to take into account the lesion size, the patient's preoperative hearing on both sides, and the relationship between the lesion and the surrounding structures, including the internal auditory canal, internal carotid artery, jugular bulb, and dura mater. There are many surgical approaches for petrous bone cholesteatoma. The frequently used approaches that do not preserve hearing were the translabyrinthine, transcochlear, and transotic approaches. The approaches that preserve hearing were the supralabyrinthine, retrolabyrinthine, and middle cranial fossa approaches [2]. The supralabyrinthine, translabyrinthine, or transotic approach assisted by an endoscope is generally considered to substitute the middle cranial fossa approach and reduce unnecessary complications [2, 3]. In the treatment of petrous bone cholesteatoma, minimally invasive surgery and complete resection of cholesteatoma have always been the direction of otologists.

However, the narrow space limits our operation, especially the supralabyrinthine space [4, 5], which is also the most common site of petrous bone cholesteatoma. In addition, the facial nerve is frequently involved. There is no consensus on how to deal with the facial nerve if the geniculate ganglion or labyrinthine facial nerve is involved. In this study, we reviewed and analyzed the cases of petrous bone cholesteatoma treated in our hospital in recent years and discussed the significance and possible risk of facial nerve transposition.

## Materials and methods

A total of 18 patients with petrous bone cholesteatoma involving the facial nerve who underwent operation via mastoid approach were treated in our hospital from November 2016 to March 2023. Patients who were lost to follow-up after surgery were excluded. The cases with incomplete facial nerves and needing nerve repair were also excluded. Patients were divided into two groups according to facial nerve transposition or nontransposition. All the patient data regarding clinical symptoms, imaging examinations, surgical methods, intraoperative findings, and follow-up results were collected and retrospectively analyzed.

Petrous bone cholesteatoma was classified according to the classification method proposed by Sanna et al. [6]. The facial nerve function was graded according to the Fisch grading system [7] and the House–Brackmann grading system [8]. The study was approved by the ethics committee of Eye & ENT Hospital of Fudan University, and informed consent was obtained from all patients participating in the study.

For a three-dimensional display of the range of cholesteatoma and to guide the operation, the computed tomography (CT) and magnetic resonance imaging (MRI) images were reconstructed using 3D Slicer (version 4.11.20210226). CT bone window sequences, diffusion-weighted imaging (DWI) sequences, and enhancement sequences of MRI were used for the reconstruction. The extension used was SlicerElastix (http://elastix.isi.uu.nl/). Detailed information about these operations can be found in the manufacturer’s instructions.

Statistical analyses were performed using SigmaPlot 14.0 (San Jose, CA, USA). Due to the small sample size, the Mann–Whitney *U* test was used to compare the values between groups. Differences were considered significant when *p* < 0.05.

## Results

### Patient characteristics and clinical symptoms

There were 13 male and five female patients. The average age at diagnosis was 36.2 years (9–71 years). The average age of patients with facial nerve transposition was 31 years (9–51 years). The average age of nontransposition patients was 38.2 years (11–71 years).

Except for two patients with hearing loss only, the other patients had hearing loss and facial paralysis symptoms to varying degrees. Facial nerve function was graded according to the Fisch grading system (Table 1). The average duration time from facial paralysis onset to operation was 13 months (1–60 months). The average duration time for patients with facial nerve transposition was 11.8 months (1–36 months), and that for nontransposition patients was 12.5 months (1–60 months). There was no statistically significant difference (*U* = 27.000; *n* = 5, 11; *p* = 1.000).

**Table 1:** Fisch facial nerve grading system before surgery

Cases	%/Points				
	At rest	Wrinkling forehead	Closing eyes	Smiling	Whistling
1	100/20	70/7	30/9	30/9	70/7
2	0/0	0/0	0/0	0/0	0/0
3	0/0	0/0	0/0	0/0	0/0
4	100/20	100/10	100/30	100/30	100/10
5	30/6	0/0	0/0	0/0	0/0
6	0/0	0/0	0/0	0/0	0/0
7	70/14	30/3	70/21	0/0	30/3
8	100/20	100/10	100/30	100/30	100/10
9	70/14	70/7	30/9	30/9	30/3
10	30/6	0/0	0/0	30/9	0/0
11	0/0	0/0	0/0	0/0	0/0
12	100/20	30/3	70/21	30/9	30/3
13	0/0	0/0	0/0	0/0	0/0
14	0/0	0/0	0/0	0/0	0/0
15	100/20	70/7	70/21	70/21	70/7
16	0/0	0/0	0/0	0/0	0/0
17	0/0	0/0	0/0	0/0	0/0
18	0/0	0/0	0/0	0/0	0/0

### CT and MRI features

**Fig. 1:**
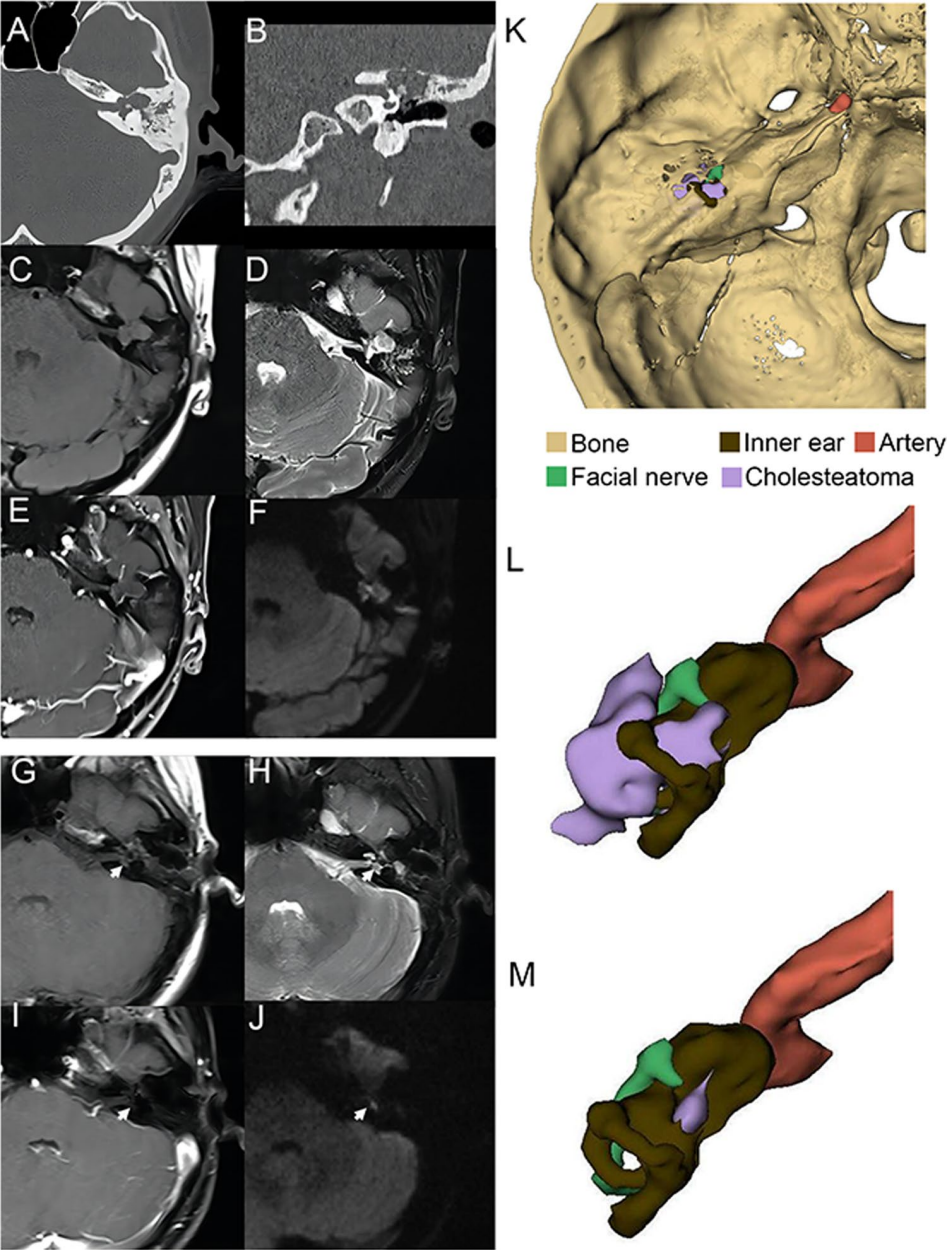
A case of cholesteatoma recurrence on imaging. **A**, **B** Left petrous bone lesion on preoperative CT involving the epitympanum, middle skull base, labyrinth, facial nerve, and internal auditory canal. **C** The mass had iso-intensity in T1W, **D** hyper-intensity in T2W, **E** annular enhancement, and **F** hyper-intensity in DWI. **G**–**J** A lesion (white arrow) appeared on MRI after the operation, characterized by iso-intensity in T1W, hyper-intensity in T2W, no enhancement, and hyper-intensity in DWI. **K** Preoperative three-dimensional reconstruction. **L** After hiding the bone, the lesion, inner ear, facial nerve, and internal carotid artery can be observed. **M** Postoperative three-dimensional reconstruction. *CT* computed tomography; *MRI* magnetic resonance imaging; *DWI* diffusion-weighted imaging; *T1W* T1-weighted; *T2W* T2-weighted

CT imaging revealed no specific manifestation. On CT, smooth bony destruction of the epitympanum, labyrinth, skull base, and petrous bone can frequently be observed (Fig. 1A, B).

On MRI, the T1-weighted signals of cholesteatoma were equal as compared with those of the cerebral white matter (Fig. 1C), and the T2-weighted (T2W) signals were high (Fig. 1D). The signals presented annular enhancement of the lesion margin by gadolinium (Fig. 1E). The DWI signals were high (Fig. 1F).

Because a three-dimensional display of petrous bone cholesteatoma can assist in judging the size of lesions and guide the operation [9], we reconstructed the CT and MRI images. In addition, the reconstruction of cholesteatoma was based on DWI sequences. We can observe the cholesteatoma and the surrounding important structures, such as the geniculate ganglion of the facial nerve, inner ear, and internal carotid artery (Fig. 1K, L).

### Treatment and facial nerve transposition

Except for one case with cholesteatoma recurrence after surgery in other hospitals, 14 cases were supralabyrinthine cholesteatoma, and three cases were massive labyrinthine, according to the classification method proposed by Sanna et al. [6]. All patients underwent operation through the retroauricular mastoid approach assisted by a microscope and an endoscope. There were 11 patients with functional bone conduction hearing before the operation; hence, we used the extended supralabyrinthine approach, that is, removing part of the surface bone of the labyrinth and cochlear with facial nerve transposition or not, to preserve the patients’ hearing. In the other six cases, we used the translabyrinthine approach in three cases of the supralabyrinthine type and the transotic approach in three cases of the massive labyrinthine type. The facial nerves of all patients were completely preserved during the operation.

**Fig. 2:**
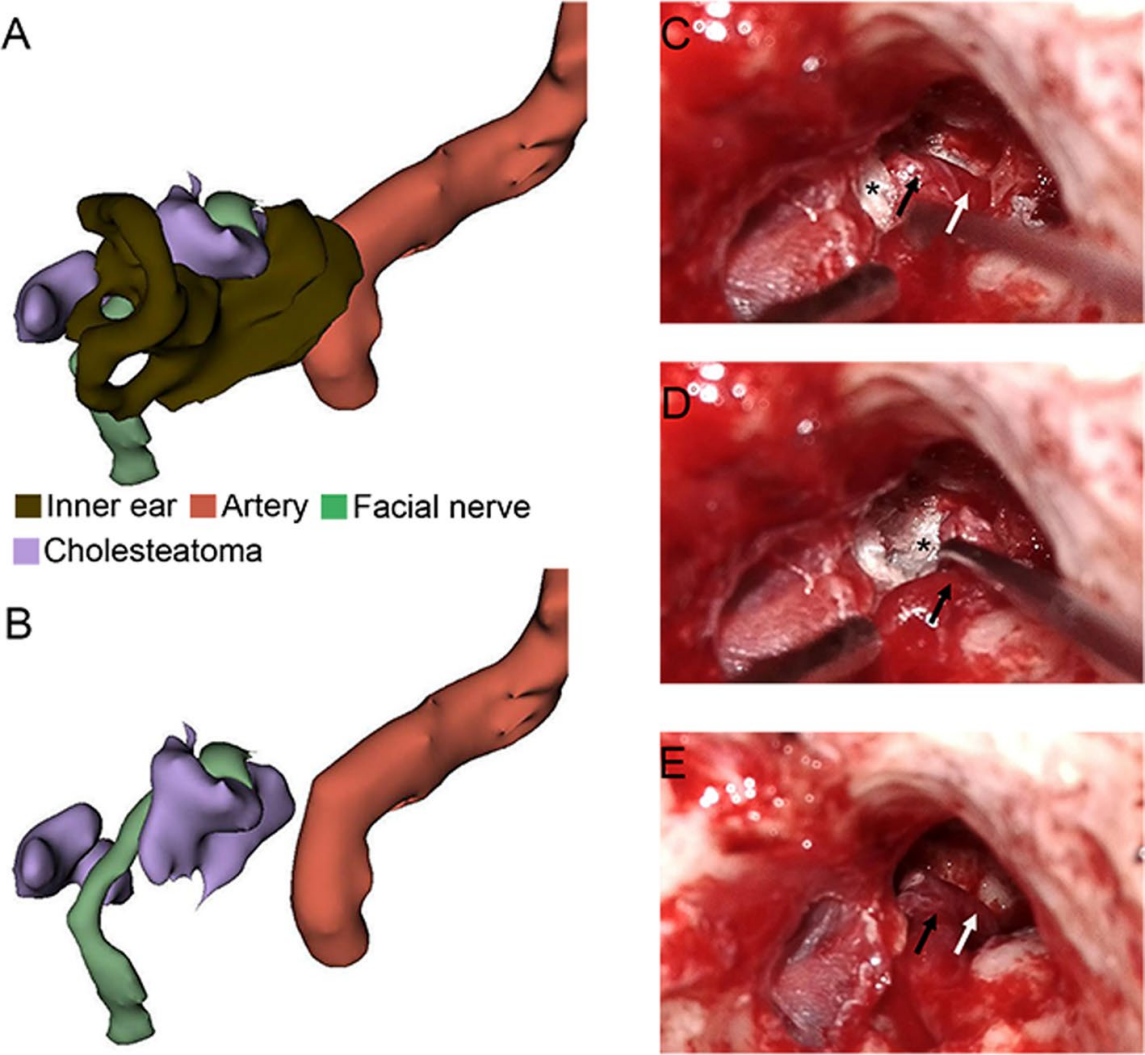
A case of facial nerve transposition. **A** Preoperative three-dimensional reconstruction showing the adjacency of cholesteatoma with the inner ear, internal carotid artery, and facial nerve. **B** After hiding the inner ear, the geniculate ganglion can be observed wrapped by cholesteatoma. **C** Under the microscope during the operation, the geniculate ganglion (black arrow), labyrinthine segment (white arrow), and cholesteatoma (asterisk) can be observed. **D** The transposition of the geniculate ganglion after the greater superficial petrosal nerve was cut off. **E** The reposition of the geniculate ganglion back to its original position

**Fig. 3:**
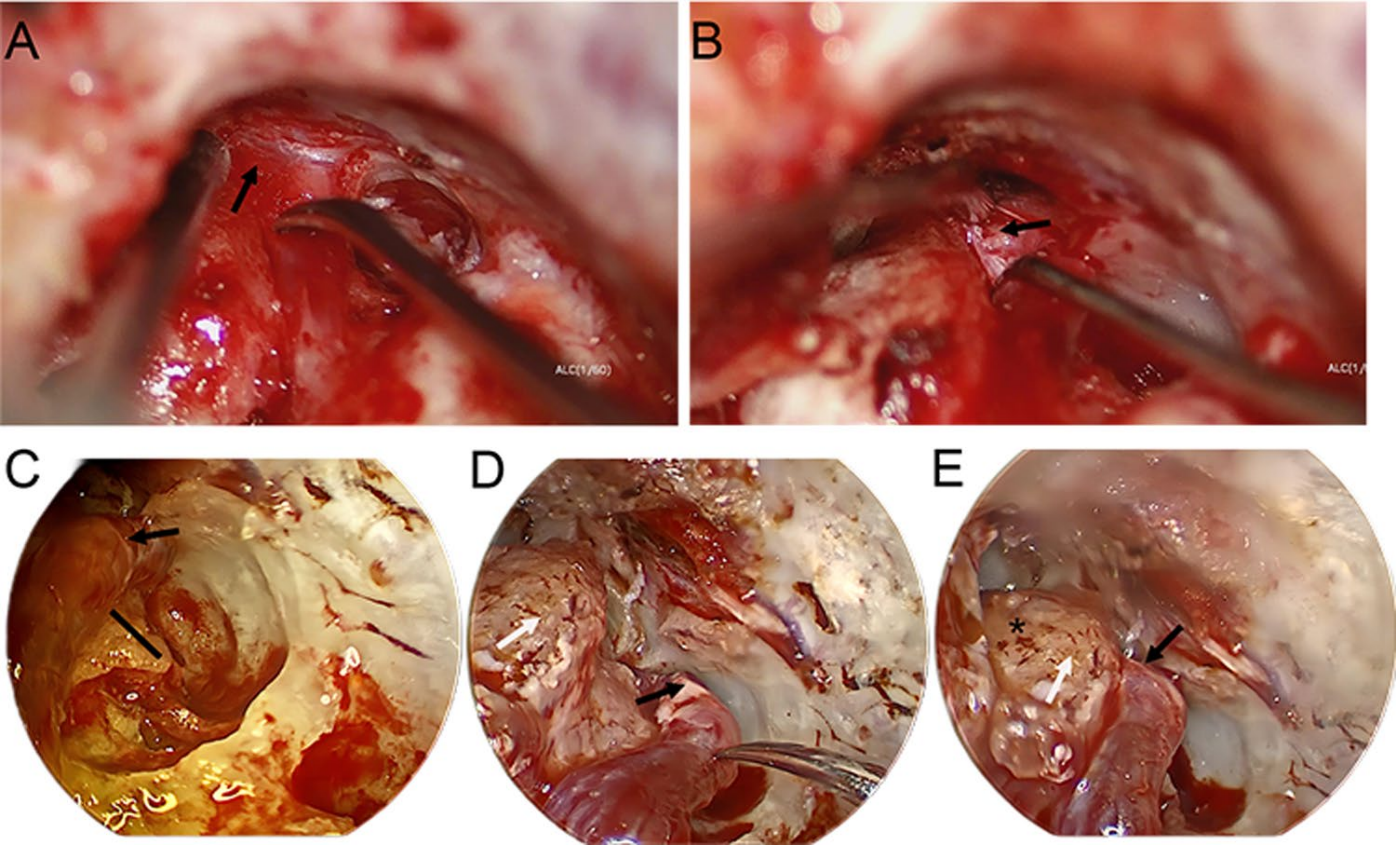
A case of difficult transposition of the facial nerve. **A** Adhesion of the geniculate ganglion (black arrow) to the dura mater. **B** The bluntly dissected geniculate ganglion (black arrow). **C** The supralabyrinthine space under the endoscope. The black line indicates the top of the vestibule. **D** Geniculate ganglion transposition (black arrow). **E** The reposition of the geniculate ganglion back to its original position. The asterisk indicates the tip of the cochlea

Temporary transposition of geniculate ganglion of facial nerve was performed in five patients, and nontransposition was performed in 13 patients. When performing facial nerve transposition, first, cut off the greater superficial petrosal nerve, move the geniculate ganglion along with the tympanic segment and labyrinthine segment, then remove the deep lesions, and put the facial nerve back to its original position (Fig. 2). In one case, the geniculate ganglion closely adhered to the dura mater. They were separated carefully first, and then, facial nerve transposition was performed (Fig. 3). The dura mater and facial nerve were completely preserved in this patient.

### Follow-up results

All patients had at least one postoperative MRI. The average follow-up time of the patients was 25.1 months (4–76 months). The average follow-up time of patients with facial nerve transposition was 32.0 months (12–76 months), and that of nontransposition patients was 22.5 months (4–43 months).

**Fig. 4:**
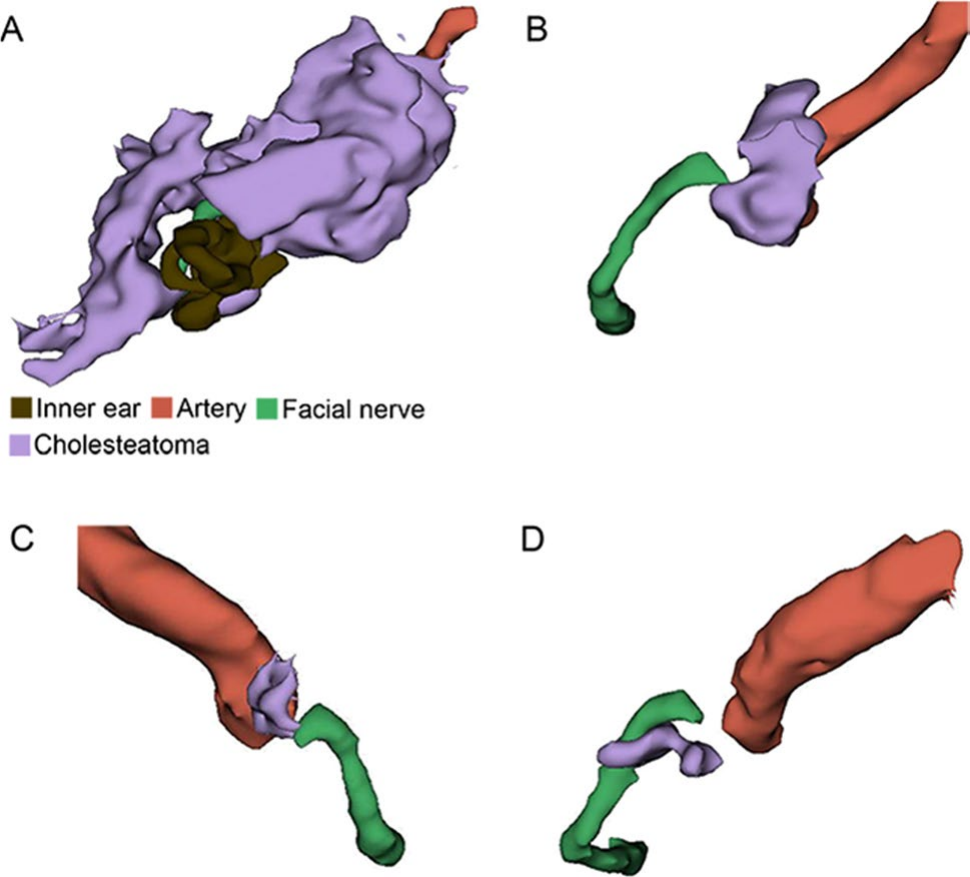
Postoperative recurrence of cholesteatoma on imaging. **A**, **B** A case of massive labyrinthine cholesteatoma. **A** The preoperative three-dimensional reconstruction; **B** 1 year after the operation of the transotic approach. **C** Image reconstruction in another case of massive labyrinthine 2 years after the operation. **D** A recurrence case 2 years after operation in another hospital. We performed facial nerve transposition in the second operation and verified the recurrence of cholesteatoma

If lesions have iso-intensity in TIW, hyper-intensity in T2W and DWI, and no obvious enhancement after contrast on MRI after the operation, we define them as cholesteatoma recurrence on imaging. Cholesteatoma recurred in three patients in the nontransposition group whereas none in the transposition group. The average recurrence time of these three patients was 15.3 months (10–24 months). Two of the three recurrence cases were of massive labyrinthine type before the operation. The locations of cholesteatoma recurrence were between the geniculate ganglion and the internal auditory canal fundus and between the geniculate ganglion and the internal carotid artery (Fig. 4A–C). In addition, the recurrence location was the same in the recurrence case of another hospital (Fig. 4D). Moreover, we performed facial nerve transposition for the case with a second operation.

We also used the House–Brackmann grading system to evaluate facial nerve function, which is more commonly used in comparison before and after surgery. Except for the previously mentioned case diagnosed with cholesteatoma recurrence that did not improve after the second operation and had always been classified as grade V, the paralysis of the other patients all had improved to varying degrees after the operation (Table 2). The cholesteatoma recurrence case had facial paralysis before the second operation for 12 months, and he was also the only one who still had dry eye and dry nose symptoms after the operation. If the paralysis does not improve after the operation, it will be classified as 0, a gradual increase will be classified as 1, and so on. The average score of patients with facial nerve transposition was 1.8 (0–4), whereas that of patients with nontransposition was 1.9 (1–3). There is no statistically significant difference in the improvement of postoperative facial paralysis between the two groups (*U* = 25.500; *n* = 5, 11; *p* = 0.859). It is worth mentioning that a 9-year-old patient with facial paralysis for 5 months had the highest score. He had an extended supralabyrinthine operation with facial nerve transposition (Fig. 3), and his facial paralysis had recovered from preoperative VI to the current II 0.5 years after the operation.

**Table 2:** Patients characteristics

Cases	Age (yrs)	FN transposition	*PTA (AC/BC, dB)		FN function (HB grades)		Approach	Follow-up (MOS)
			Pre	Post	Pre	Post		
1	57	No	−/−	−/−	III	II	L	30
2	40	No	51/23	65/30	VI	V	ES	24
3	29	No	86/31	117/52	VI	III	ES	27
4	53	No	73/38	80/40	I	I	ES	43
5	23	No	46/13	40/16	V	IV	ES	36
6	27	No	51/33	65/35	VI	IV	ES	33
7	70	No	−/−	−/−	III	I	O	12
8	27	No	70/30	25/15	I	I	ES	35
9	71	No	91/55	100/61	III	II	ES + A	18
10	11	No	28/15	25/10	IV	I	ES	4
11	43	No	−/−	−/−	V	II	O	24
12	29	No	−/−	−/−	III	I	O	12
13	27	No	43/18	22/10	VI	III	ES	12
14	51	Yes	−/−	−/−	V	V	S	34
15	23	Yes	73/41	65/35	II	I	ES	17
16	9	Yes	43/13	35/20	VI	II	ES	12
17	28	Yes	−/−	−/−	VI	IV	L	21
18	44	Yes	−/−	−/−	VI	IV	L	76

*PTA, the average values of pure tone audiometry at 500 Hz, 1 kHz, and 2 kHz

*AC* air conduction; *BC* bone conduction; −/− preoperative BC > 80 dB; *YRS* years; *MOS* months; *FN* facial nerve; *Pre* preoperative; *Post* postoperative; *L* translabyrinthine; *O* transotic; *ES* extended supralabyrinthine; *A* infratemporal fossa type A approach; *S* second operation

In addition, the hearing was evaluated half a year after surgery. All patients who underwent the supralabyrinthine approach surgery had their bone conduction hearing preserved (the hearing threshold of bone conduction decreases or increases by less than 10 dB), except for one patient whose bone conduction hearing decreased from 31 to 52 dB (Table 2).

## Discussion

In petrous bone lesions, asymmetric pneumatization and effusion are the most common, followed by cholesterol granuloma and then cholesteatoma. Petrous bone cholesteatoma is a type of expansile epidermoid lesion with slow growth, accounting for 4–9% of all petrous lesions, and its incidence is 1/10 of cholesterol granuloma [10, 11].

Petrous bone cholesteatoma may be congenital, acquired, or iatrogenic [1]. Patients with petrous bone cholesteatoma have no characteristic symptoms. The main symptom is hearing loss, followed by facial paralysis of differing severity. Other symptoms include dizziness, tinnitus, aural fullness, and otalgia. When combined with infection, there may be otorrhea or neck abscess. Very few cases will involve the petrous apex and develop Gradenigo’s syndrome, namely, diplopia, periorbital numbness, and retrobulbar pain [1, 12, 13]. The diagnosis of petrous bone cholesteatomas mainly depends upon imaging. On CT, cholesteatomas show bone destruction with smooth edges and annular enhancement. On MRI, the lesions show a low or equal signal on the T1-weighted sequence and no enhancement by gadolinium, show a high signal on the T2W sequence, and show limited diffusion and a high signal on DWI sequence [13, 14]. MRI, especially DWI, is also a useful and reliable tool for follow-up cholesteatoma surgery [15, 16].

Although the incidence is low, it is destructive [17]. In 1993, Sanna divided petrous bone cholesteatomas into five types: supralabyrinthine, infralabyrinthine, massive labyrinthine, infralabyrinthine-apical, and apical [6]. The supralabyrinthine type is the most common in clinical practice. The supralabyrinthine space refers mainly to the enclosed area between the middle cranial fossa plate, the geniculate ganglion, the vestibular and cochlear surface, and the horizontal and superior semicircular canals [4, 12].

In the supralabyrinthine and massive labyrinthine cases, the geniculate ganglion, labyrinthine segment of the facial nerve, and even the deeper part of the petrous bone are often invaded by cholesteatoma. In these cases, there is no consensus on how to deal with the facial nerve. Theoretically, nerve transposition can help to obtain a better surgical vision, which is conducive to clearing the lesions medial to the geniculate ganglion. However, facial nerve transposition is a challenging operation, necessitating the sacrifice of the greater superficial petrosal nerve, and there is a risk of facial nerve injury.

In the patients with facial nerves involved by cholesteatoma but without facial nerve transposition, we found three patients with recurrence during MRI follow-up after surgery. Two of the three cases were of massive labyrinthine type before operation. The recurrence sites of these cases were all areas medial to the geniculate ganglion (Figs. 1 and 4). It was the same in the recurrence case of another hospital (Fig. 4). In patients with facial nerve transposition, no recurrence was found during follow-up. These results are in line with expectations. Because of the visual field obstruction of the facial nerve, cholesteatoma epithelium may remain.

In patients with facial nerve transposition, the worrying problems are whether the facial nerve will be damaged and whether the amputation of the greater superficial petrosal nerve will cause postoperative ocular and nasal discomfort. In previous reports on lateral skull base surgery, such as paraganglioma, facial nerve transposition was often accompanied by a significant decrease of facial nerve function [18]. During the follow-up, we found that, except for the previously mentioned case that did not improve after the second operation, the facial paralysis of other patients all had improved to varying degrees. There was no significant difference in the improvement of postoperative facial paralysis between the facial nerve transposition group and the nontransposition group. The improvement of facial paralysis after the operation was related to the duration and degree of facial paralysis and the age of patients before the operation. In addition, there was no obvious eye and nose discomfort in the patients with postoperative facial paralysis of grade IV or better, regardless of facial nerve transposition. Dry eye and dry nose symptoms were only found in one patient with postoperative facial paralysis of grades V. In lateral skull base surgery, the facial nerve requires extensive permanent transposition, resulting in significant surgical trauma and blood supply disruption. However, in our case, it mainly involves the temporary transposition of the geniculate ganglion. This should be the reason for the different results. Once the petrous cholesteatoma is removed, the facial nerve is no longer compressed and its function may be restored. De Brito et al. also reported that improved partial facial nerve transposition or slightly displacing it can significantly improve postoperative facial nerve function during lateral skull base surgery [19].

In patients where cholesteatoma is medial to the geniculate ganglion and is difficult to remove, a routine transposition of the geniculate ganglion should be performed. In addition, poor preoperative facial function implies severe facial nerve injury or close relationship between cholesteatoma and facial nerve, which is also an indication for facial nerve transposition. The operator should be an experienced otologist, be familiar with the anatomy of the facial nerve in the temporal bone and be patient enough. In some complex cases, facial nerve transposition may be very challenging, such as the adhesion of the geniculate ganglion to the dura mater. In this case, blunt separation is feasible.

## Conclusion

The combination of microscopy and endoscopy can help expand the surgical vision, which is beneficial for lesion clearance. For cases of petrous bone cholesteatoma where facial nerve is obviously involved, facial nerve transposition should be performed. Temporary facial nerve transposition will not affect the postoperative nerve function, will not cause postoperative eye and nose discomfort, and can reduce the possibility of cholesteatoma recurrence in the regions medial to the geniculate ganglion. Patients with massive labyrinthine have a high probability of recurrence after surgery, so facial nerve transposition can be performed routinely in these patients.

## Data Availability

The datasets used and/or analyzed during the current study are available from the corresponding authors on reasonable request.
